# Relationship between Frontal Gap and Postoperative Stability in the Treatment of Mandibular Prognathism

**DOI:** 10.1155/2016/7046361

**Published:** 2016-09-27

**Authors:** Yu-Chuan Tseng, Kun-Jung Hsu, Ker-Kong Chen, Ju-Hui Wu, Chun-Ming Chen

**Affiliations:** ^1^Department of Orthodontics, Kaohsiung Medical University Hospital, Kaohsiung, Taiwan; ^2^Graduate Institute of Dental Sciences, College of Dental Medicine, Kaohsiung Medical University, Kaohsiung, Taiwan; ^3^Department of Family Dentistry, Kaohsiung Medical University Hospital, Kaohsiung Medical University, Kaohsiung, Taiwan; ^4^Department of Oral Hygiene, College of Dental Medicine, Kaohsiung Medical University, Kaohsiung, Taiwan; ^5^Department of Oral and Maxillofacial Surgery, Kaohsiung Medical University Hospital, Kaohsiung, Taiwan

## Abstract

*Objectives*. To investigate the correlation between frontal gaps and skeletal stability after intraoral vertical ramus osteotomy (IVRO) for correction of mandibular prognathism.* Materials and Methods*. Thirty-three patients with frontal gaps after IVRO-based mandibular prognathism correction were included. Three lateral and frontal cephalometric radiographs were obtained: preoperatively (T1), immediately postoperatively (T2), and 2 years postoperatively (T3). Two linear measurements (menton [Me] and frontal gap) were compared from T1 to T3 (T21: immediate surgical changes; T32: postoperative stability; T31: 2-year surgical change). Data were analyzed using Pearson's correlation coefficient and multiple linear regression.* Results*. The T21 mean surgical horizontal change in the Me position was 12.4 ± 4.23 mm. Vertically, the mean downward Me movement was 0.6 ± 1.73 mm. The mean frontal gaps were 4.7 ± 2.68 mm and 4 ± 2.48 mm in the right and left gonial regions, respectively. Postoperative stability (T32) significantly correlated with the amount of setback. Frontal gaps did not have a significant effect on postoperative stability. However, multiple regression model (*R* ^2^ = 0.341, *P* = 0.017) showed value predictability, especially in the amount of setback.* Conclusion*. Frontal gaps occur after IVRO but have no significant effect on long-term postoperative skeletal stability. The primary risk factor for postoperative relapse remains the amount of mandibular setback.

## 1. Introduction

Skeletal discrepancy in the maxilla or mandible can be due to morphological malformation or asymmetry and tends to induce significant malocclusion and dentofacial deformity [1]. Mandibular prognathism refers to the prominent protrusion of the lower third portion of the facial skeleton. This facial pattern is commonly seen among siblings and parents because of its strong heritability [2].

Combined orthodontic treatment and orthognathic surgery has been advocated as the major approach for correction of mandibular prognathism. Intraoral vertical ramus osteotomy (IVRO) and sagittal split ramus osteotomy (SSRO) are the two main surgical approaches for treating prognathic deformity of the mandible. The main advantage of IVRO is that it has a markedly lower incidence of nerve damage than SSRO [3, 4]. The major disadvantage of IVRO is that the patient must be subjected to intermaxillary fixation (IMF) to immobilize both segments and thus must remain on a liquid diet until removal of the IMF device. Even Nihara et al. [5] reported only 1-week IMF by 4 monocortical screws for IVRO. However, we still performed 6-week IMF to avoid the movement of both segments during bone healing. Nevertheless, to avoid postoperative lip numbness, we advocate the use of IVRO rather than SSRO for the treatment of mandibular prognathism.

A frontal gap is a transverse horizontal space that occurs between the lateral proximal segment (condyle-bearing) and the medial distal segment (tooth-bearing) as they overlap. As with SSRO, osteosynthesis fixation can eliminate frontal gaps. The surgical site of IVRO, which involves the gonial region, is different from that targeted in SSRO. After mandibular setback, it is easy to determine whether frontal gaps have developed in IVRO patients due to the lack of fixation between the proximal and distal segments. Therefore, a frontal gap only occurs in IVRO, but not in SSRO. A frontal gap can influence bone healing and remodeling, and differences in the gap may correlate with postoperative stability. Nevertheless, the influence of frontal gaps on postoperative relapse has not been thoroughly evaluated. Therefore, the present study assessed how long-term stability is affected by frontal gaps and the amount of mandibular setback in patients with mandibular prognathism who were treated with IVRO.

## 2. Materials and Methods

### 2.1. Patients and Measurements

Thirty-three patients who had only undergone IVRO for correction of mandibular prognathism at the Department of Oral and Maxillofacial Surgery of the Kaohsiung Medical University Hospital were enrolled. All the patients were operated on using a modified IVRO technique [4]. Patients were excluded if they had facial asymmetry, a history of facial bone trauma, or congenital craniofacial anomalies. Postoperatively, IMF was maintained for 6 weeks. No fixation between the proximal and distal segments was performed.

**Figure 1:**
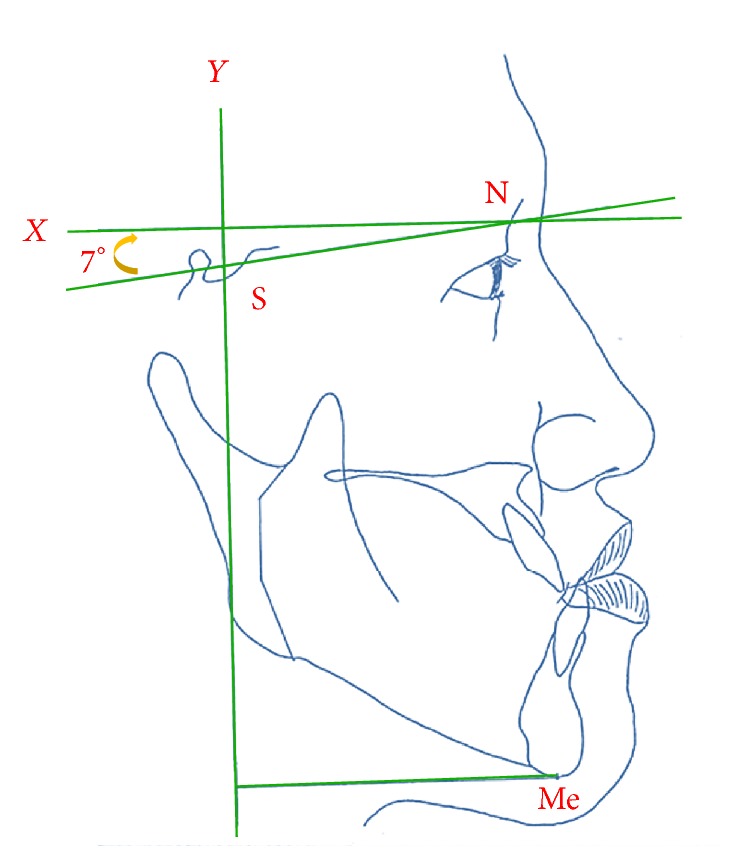
*X*-axis: constructed by drawing a line through nasion 7° up from SN line. *Y*-axis: constructed by drawing a line through sella (S) perpendicular to the *X*-axis. Me: the most inferior point on the mandibular symphysis.

**Figure 2:**
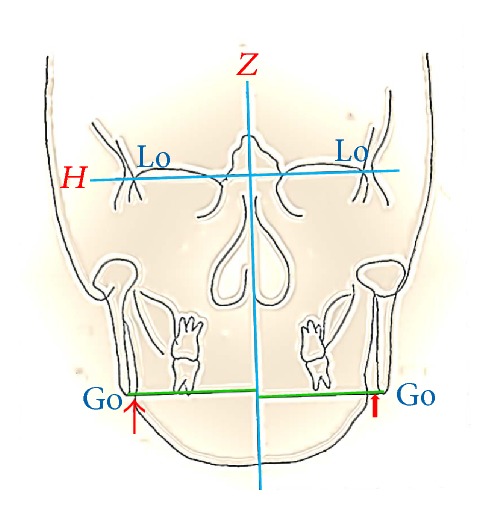
*H* line: horizontal reference line; *Z* line: midsagittal plane. Landmarks: gonion (Go) and lateroorbitale (Lo). Narrow red arrow: right side frontal gap; wide red arrow: left side frontal gap.

Patients were investigated by serial lateral cephalograms (preoperatively [T1], 48 h after surgery [T2], and 2 years after surgery [T3]). The T2 posteroanterior (PA) cephalogram was used to measure frontal gaps. The landmarks chosen in the lateral cephalogram included the sella (S), nasion (N), and menton (Me). In addition, the gonion (Go) and lateroorbitale (Lo) were selected as landmarks on the PA cephalogram. The horizontal and vertical reference lines (*X* line and *Y* line) were constructed for analysis. In lateral cephalograms, the *X* line was set up at 7 degrees superior to the sella-nasion line, passing through point N [6]. The *Y* line was constructed perpendicularly to the *X* line through landmark S (Figure 1). In PA cephalograms, a horizontal reference line was established as the line running through the bilateral Lo landmarks, and a vertical reference line was defined as the *Z* line, which was at a right angle to the *H* line in the midsagittal plane (Figure 2).

The linear measurements for this study included the distance from the Me to the reference lines and the frontal gap (distance between the horizontal plane and Go, intersecting the lateral border of the ramus). Two linear measurements (menton [Me] and frontal gap) were compared from T1 to T3 (T21: immediate surgical changes; T32: postoperative stability; T31: 2-year surgical change). The frontal gap was clarified on the T2 images. This study was approved by the ethics committee of Kaohsiung Medical University Hospital.

### 2.2. Statistical Analysis

Paired *t*-tests were used to compare the differences between the T1 and T3 periods. Pearson's correlation coefficient was calculated between Me T32 (the 2-year postoperative change in the horizontal direction) and related variables. Multiple regression analysis was performed to clarify the factors contributing to postoperative relapse. Data were analyzed with SPSS 20 and the significance level was set as *P* < 0.05.

Fifteen X-rays were randomly measured twice by Chun-Ming Chen after a 10-day interval. Systematic errors were evaluated using a paired *t*-test for frontal gap, and no significant difference (*P* = 0.488) was observed. Accidental errors were calculated using the Dahlberg formula, which is expressed as follows:

(1) $$\begin{array}{c} \text{accidental errors}=\sqrt{\frac{\sum d^{2}}{2n}}, \end{array}$$

where *d* represents the difference between the 2 sets of data and *n* represents the number of measurements. Accidental errors (0.123 mm on average) were shorter than 0.5 mm, thus indicating the sufficient accuracy of the measurements.

## 3. Results

There were 20 female and 13 male patients, with a mean age of 20.4 years (range: 17–34 years). The average surgical change (T21) at the Me was 12.4 ± 4.23 mm (*P* < 0.0001) backward; this difference was statistically significant (Table 1). In the vertical direction, the Me moved significantly downward, by 0.6 ± 1.73 mm (*P* = 0.048). The frontal gaps were 4.7 ± 2.68 mm (range: 0.5–11) and 4 ± 2.48 mm (range: 0.5–10) in the right and left gonial regions; these differences were not statistically significant (*P* = 0.079). The paired *t*-test revealed no significant relapse of the Me (T32) in the horizontal and vertical directions: only 1 ± 3 mm (*P* = 0.068) forward movement and 0.5 ± 1.96 mm (*P* = 0.153) upward movement.

Horizontal relapse (T32) was significantly (*P* = 0.003) correlated with the amount of mandibular setback (T21) and vertical movement (T32 and T31). Vertical relapse (T32) was significantly correlated with horizontal changes (T21 and T32) and vertical changes (T21 and T31). The degree of upward relapse of the Me showed a trend toward significance, but it was too small to have a clinical effect (Table 2).

In Table 3, there was no statistically significant correlation between postoperative stability and the extent of the frontal gap. However, multiple regression analysis showed that the frontal gap was useful for prediction of horizontal relapse (T32) (*R* ^2^ = 0.341, *F* = 3.616, *P* = 0.017), using the following equation: horizontal relapse (Me T32) = −1.833 − 0.283 × horizontal (Me T21) − 0.259 × vertical (Me T21) + 0.157 × right frontal gap − 0.401 × left frontal gap. The amount of setback (Me T21) was significantly (*P* = 0.022) correlated with horizontal relapse (Me T32).

## 4. Discussion

Orthognathic surgery aims to achieve facial esthetics and to improve masticatory function. These goals can be achieved by eliminating facial disharmony and reconstructing the facial skeleton to achieve balanced proportions. Long-term postoperative stability in correcting mandibular prognathism plays an important role in maintaining treatment outcome. Previous studies [7–9] have revealed that postoperative stability is influenced by several factors. First, the postsurgical stability of the jawbones is closely related to ongoing growth in patients. Although most orthognathic surgeries are performed in postpubertal patients, this does not guarantee that no further growth and development are taking place in an individual patient. It is highly likely that ongoing growth can induce recurrence of skeletal disharmony, resulting in an open bite.

Surgical modalities used to correct mandibular prognathism can also influence postsurgical stability. Several studies [8–12] have investigated the correlation between contributing factors and postsurgical stability, among which the amount of setback had the strongest effect on postoperative stability. The above investigations all indicated that, regardless of which parameter (such as B point, Pog, Me, Ii, or overjet) was used to define the amount that the mandible was set back by horizontally after surgery, the extent of mandibular movement always fell within 10 mm. Compared to the results of previous studies that used IVRO without IMF [9–13], the immediate postsurgical changes in the mandible in the present study revealed an average setback of 12.4 mm for the Me. Thus, the average amount of mandibular movement noted in our study far exceeded that in previous reports. Even in the absence of postoperative obstructive sleep apnea, the preoperative and postoperative dimensions of the pharyngeal airway space should be considered and evaluated. Moreover, the correlation between the frontal gap and temporomandibular joint disorder should be investigated in the future.

Abeltins et al. [13] performed IVRO in 30 patients with a mean 4.4 mm setback at the B point. At the 1-year postoperative follow-up, the amount of relapse was significant (1.2 mm). Ohba et al. [14] assessed the 1-year skeletal stability in 16 patients treated for mandibular prognathism by IVRO. The Me setback was 6.4 mm; at 1-year postoperatively, the setback was still 4.18 mm backward, and the difference was not statistically significant. The findings of our previous report [1] were similar to those of Ohba et al. [14]. At the 1-year follow-up, our patients exhibited a 0.1 mm relapse, which was not statistically significant. According to a previous report, the mandible could have moved forward or backward by the 1-year follow-up. Therefore, we recommend that investigation of the postoperative stability of IVRO requires a follow-up of at least 2 years. Moreover, to evaluate postoperative stability, computed tomography may be better than cephalography for assessing the progress of bone healing.

In the present study, the Me (T32) showed a 1 mm (8.1%) horizontal relapse over the follow-up period, which was not statistically significant. This small postoperative relapse can be attributed to (1) complete stripping off of the medial pterygoid and masseter muscles and (2) excision of the inferior proximal segment. This approach allows a greater degree of mandibular setback and reduces the amount of stretching of the pterygomandibular sling. In our multiple regression model (*R* ^2^ = 0.341, *P* = 0.017), the amount of setback (Me T21) allowed prediction of postoperative relapse (Me T32); thus, the amount of setback had a significant effect (*P* = 0.022) on postoperative stability. Even though there was no correlation between the frontal gap and postoperative stability, some other factors, such as condylar drift and masticatory force, should be considered as possible contributors to postoperative relapse.

To evaluate the factors contributing to the stability after SSRO, Guglielmi et al. [15] stressed the importance of assessing changes in the gonial angle. In particular, IVRO involves cutting through the gonial region and overlapping these divided segments, and this change was more than that achieved with SSRO. Due to differences in the surgical technique, the dimensions of the frontal gap between the proximal and distal segments of the mandible are significantly different between SSRO and IVRO [16]. In our previous study [17], the intergonial distance was significantly increased with the IVRO technique. Despite the fact that the frontal gaps induced by IVRO were marked (4.7 mm on the right side and 4 mm on the left side), our study did not show any significant correlation between the extent of the frontal gap and the long-term postoperative relapse.

The bone healing and remodeling processes after IVRO differ from those after SSRO. In the IVRO technique, a frontal gap of more than 1 mm can easily occur between the proximal and distal segments. In particular, when the distal segment is set back further, it could push the proximal segment more laterally. Therefore, a larger than expected frontal gap can occur. This is easily detected by postoperative PA radiography. Without intersegment fixation, condylar sag and drooping would be greater in IVRO than in SSRO. In other words, some mobility between the proximal and distal segments remains after removal of the IMF, because the calluses at this stage are still soft and the strength of the link between the proximal and distal segments is not sufficient to resist the biting force. Once masticatory activity gradually increases, any improper orthodontic traction, such as use of interarch elastics, may potentially produce undesired movement and consequently induce mandibular relapse.

In conclusion, frontal gaps between the proximal and distal segments, induced by IVRO surgery, did not demonstrate any marked influence on long-term postoperative stability. However, there was a greater chance of postoperative relapse with an increase in mandibular setback.

## Figures and Tables

**Table 1:** Summary of menton (Me) and frontal gap in T21, T32, and T31.

Variable	Mean	SD	*P* value
Me (horizontal, mm)			
T21	−12.4	4.23	<0.001^*∗*^
T32	1.0	3.00	0.068
T31	−11.4	3.78	<0.001^*∗*^
Me (vertical, mm)			
T21	0.6	1.73	0.048^*∗*^
T32	−0.5	1.96	0.153
T31	0.1	1.59	0.664
Frontal gap T2 (mm)			
Right gap	4.7	2.68	0.079
Left gap	4.0	2.48	

T21: immediate surgical changes; T32: postoperative stability; T31: 2-year surgical change.

Significant ^*∗*^ *P* < 0.05.

**Table 2:** Postoperative stability (T32) by Pearson's correlation test.

Variable	Me horizontal T32		Me vertical T32	
	Coefficient	*P* value	Coefficient	*P* value
Me (horizontal, mm)				
T21	−0.494	0.003^*∗*^	0.394	0.023^*∗*^
T32	1		−0.650	<0.001^*∗*^
T31	0.240	0.178	−0.075	0.679
Me (vertical, mm)				
T21	0.303	0.087	−0.637	<0.001^*∗*^
T32	−0.650	<0.001^*∗*^	1	
T31	−0.473	0.005^*∗*^	0.540	0.001^*∗*^
Frontal gap T2 (mm)				
Right gap	−0.001	0.998	−0.031	0.863
Left gap	−0.296	0.094	0.249	0.162

T21: immediate surgical changes; T32: postoperative stability; T31: 2-year surgical change.

Significant ^*∗*^ *P* < 0.05.

**Table 3:** Prediction of postoperative stability (horizontal T32) by the multiple regression analysis.

	Unstandardized coefficients		Standardized coefficients		
	*B*	SE	*β*	*t*	*P*
Constant	−1.833	1.703		1.076	0.291
Me horizontal T21	−0.283	0.117	−0.399	−2.426	0.022^*∗*^
Me vertical T21	0.259	0.280	0.150	0.924	0.363
Right gap	0.157	0.222	0.140	0.707	0.485
Left gap	−0.401	0.239	−0.331	−1.673	0.105

*B*: regression coefficient; SE: standard error; *β*: standardised regression coefficient; *t*: obtained *t*-test value; *P*: obtained significance value.

T21: immediate surgical changes; T32: postoperative stability.

Significant ^*∗*^ *P* < 0.05.
